# Gamma radiation induces hydrogen absorption by copper in water

**DOI:** 10.1038/srep24234

**Published:** 2016-04-18

**Authors:** Cláudio M. Lousada, Inna L. Soroka, Yuriy Yagodzinskyy, Nadezda V. Tarakina, Olga Todoshchenko, Hannu Hänninen, Pavel A. Korzhavyi, Mats Jonsson

**Affiliations:** 1 Division of Materials Technology, Department of Materials Science and Engineering, KTH Royal Institute of Technology, SE-100 44 Stockholm, Sweden; 2 School of Chemical Science and Engineering, Applied Physical Chemistry, KTH Royal Institute of Technology, SE-100 44 Stockholm, Sweden; 3 School of Engineering, Aalto University, Puumiehenkuja 3, 02150 Espoo, Finland; 4 The NanoVision Centre, School of Engineering and Materials Science, Queen Mary University of London, Mile End, London E1 4NS, United Kingdom; 5 Experimentelle Physik III, Physikalisches Institut and Wilhelm Conrad Röntgen - Research Centre for Complex Material Systems, Universität Würzburg, Am Hubland, D-97074 Würzburg, Germany

## Abstract

One of the most intricate issues of nuclear power is the long-term safety of repositories for radioactive waste. These repositories can have an impact on future generations for a period of time orders of magnitude longer than any known civilization. Several countries have considered copper as an outer corrosion barrier for canisters containing spent nuclear fuel. Among the many processes that must be considered in the safety assessments, radiation induced processes constitute a key-component. Here we show that copper metal immersed in water uptakes considerable amounts of hydrogen when exposed to γ-radiation. Additionally we show that the amount of hydrogen absorbed by copper depends on the total dose of radiation. At a dose of 69 kGy the uptake of hydrogen by metallic copper is 7 orders of magnitude higher than when the absorption is driven by H_2_(g) at a pressure of 1 atm in a non-irradiated dry system. Moreover, irradiation of copper in water causes corrosion of the metal and the formation of a variety of surface cavities, nanoparticle deposits, and islands of needle-shaped crystals. Hence, radiation enhanced uptake of hydrogen by spent nuclear fuel encapsulating materials should be taken into account in the safety assessments of nuclear waste repositories.

## Introduction

Nuclear power is often argued to be a fossil-free alternative in the global spectrum of electricity generation^1^,^2^. The safety of operating nuclear power plants is usually of main concern in discussions comparing different energy production techniques. However, at present, one of the most difficult issues to tackle is the long-term safety of repositories for radioactive waste originating from nuclear power plants, in particular the spent nuclear fuel^3^. The safety of such repositories must be guaranteed for time periods longer than the history of *homo sapiens*^4^. The safety relies on a series of natural and engineered barriers preventing the highly radioactive material from migrating into the biosphere^5^. In some repository concepts, metallic copper has been proposed as an outer corrosion resistant barrier on canisters containing the spent nuclear fuel^6^,^7^,^8^. As for the other barrier materials, studies of processes that have potential impact on the long-term integrity of the copper layer have been conducted both in laboratories and in the field under repository conditions^9^. A rather unique feature of the copper used in spent nuclear fuel canisters is that it will be exposed to ionizing radiation^10^,^11^,^12^,^13^,^14^. Because of this it is of vital importance to elucidate the effects of ionizing radiation on copper and on the interface between copper and surrounding matter.

**Figure 1:**
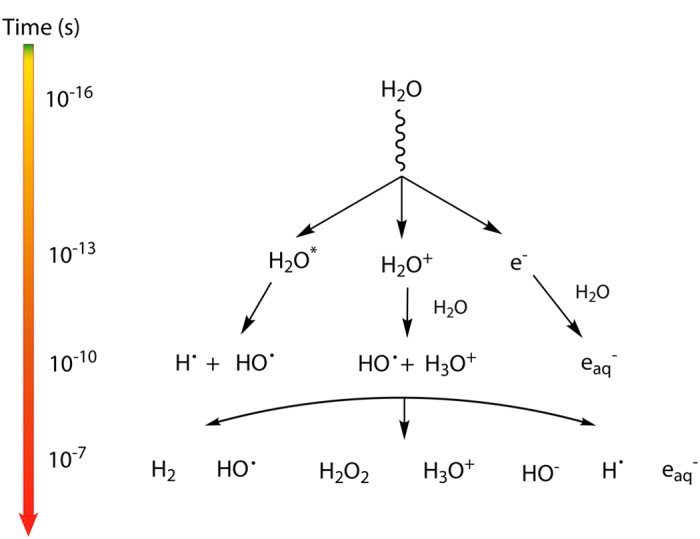
Time scale of events in water radiolysis leading to the primary products: H_2_; HO^•^; H_2_O_2_; H_3_O^+^; HO^−^; H^•^ and e_aq_^−^.

The consequences of the exposure of many homogeneous systems to ionizing radiation are well-known on the basis of both experimental and theoretical studies performed over a period close to a century^15^. However, most systems of practical relevance are not homogeneous. In fact, one of the most crucial and thereby also interesting components of a system from a performance perspective is the interface between two phases. In nuclear technology, interfaces exposed to ionizing radiation constitute a key-point in any safety assessment and a common theme is the impact of radiation-induced corrosion in nuclear power plants, nuclear fuel reprocessing plants and repositories for radioactive waste^16^,^17^. In spite of the obvious importance of interfacial radiation chemistry, surprisingly, little is known about underlying mechanisms.To describe the radiation chemistry at an interface it is necessary to have information about: the homogeneous radiation chemistry of the two phases in contact; the energy deposition in the system; the yield of radiolysis products; and the reactivity of radiolysis products at the interface between the two homogeneous phases. γ-radiolysis of liquid water leads to a number of reactive species that are formed on different time scales^18^ as depicted in Fig. 1. The radiation chemical yield (G-value) of a product of γ-radiolysis of water may be affected by the presence of chemical species or materials in the aqueous medium^19^. Several studies have shown that the yield of the aqueous radiolysis product H_2_ can be significantly higher in water layers close to an oxide surface when compared to pure bulk water^20^,^21^. The magnitude of this effect depends on the nature of the oxide. However, no convincing mechanistic explanation that accounts for these observations has been given. In addition to the somewhat puzzling observations regarding the radiation chemical yield of H_2_, a number of studies on the interactions between other aqueous radiolysis products and oxide surfaces have been presented. It has been shown that H_2_O_2_ is catalytically decomposed^22^ to O_2_ and H_2_O via the intermediate formation of hydroxyl radicals on most oxide surfaces, and that the hydroxyl radical has a high affinity for oxide surfaces^23^. Additionally, under certain conditions, the decomposition of H_2_O_2_ at oxide surfaces can also lead to the formation of H_2_^24^.

It is known that hydrogen has undesirable effects on the life-time and performance of many metallic materials^25^. Hydrogen can be absorbed by metals via the diffusion of H-atoms through preferred specific pathways that have low energy barriers for H-atom migration^26^. Upon radical recombination of the H-atoms, H_2_ is formed and its effects on materials structure can be considerable. Such effects are the result of changes on vacancy-vacancy interactions^27^ which ultimately leads to the formation of internal cavities and other defects that are accompanied by the deterioration of the physical-chemical properties of the materials^28^. Furthermore, γ-radiation can induce changes in nuclear spin states of H_2_ and lead to the conversion of para- to ortho-hydrogen^29^. It is known that changes in the nuclear spin state of H_2_ cause changes in its magnetic properties and this affects its interactions with metals leading to increased stability of hydrogen at some positions in the copper lattice^30^. Additionally, γ-radiation is known to cause low temperature annealing of metals^31^. The fact that γ-radiation is capable of inducing structural changes in the bulk material^32^ and interface with water^21^—including corrosion^33^,^34^,^35^—lead us to develop this study, which is aimed at understanding whether γ-radiation is capable of triggering the uptake of hydrogen by high-purity metallic copper in aqueous medium. As shown above, one of the radiolysis products of water is hydrogen^36^. Hence, the undesirable effects that hydrogen has on copper could potentially hamper the barrier properties.

### Radiation-induced hydrogen charging of copper metal in water

**Figure 2:**
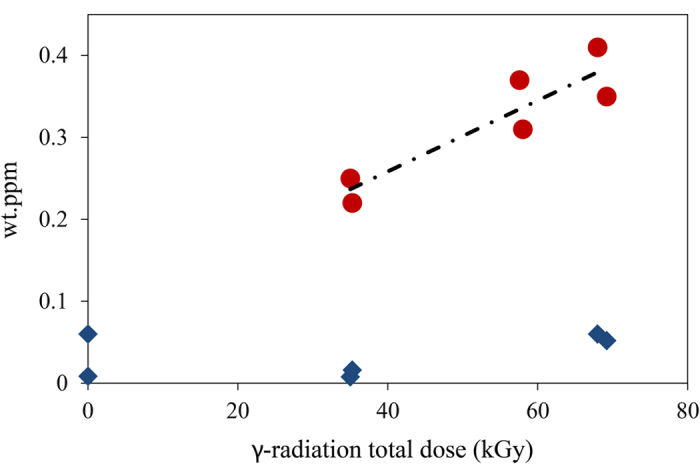
Amounts of H_2_ (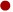) and H_2_O (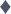) measured in samples of copper metal irradiated in water as a function of the total dose of γ-radiation deposited (*D*) (kGy). The measurements of H_2_ and H_2_O were performed after irradiation. Each data point corresponds to a different irradiation experiment. Both sets of data are normalized for the background values. The linear regression is given by: *y* = 4 · 10^−6^*x* + 0.085; *R*^2^ = 0.827.

**Figure 3:**
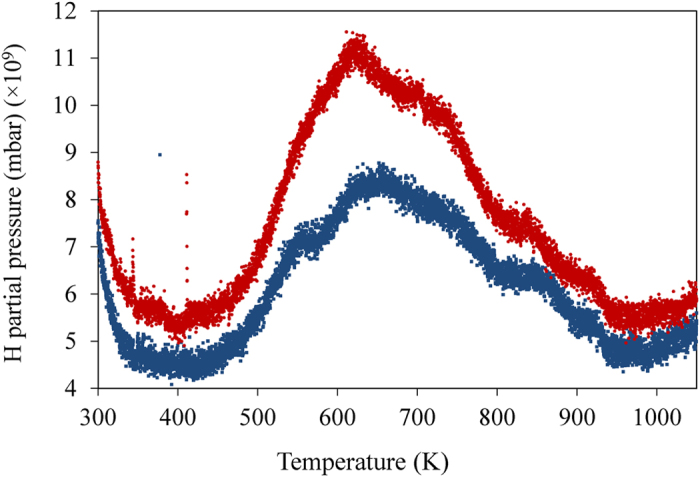
Plot of the H partial pressure in the UHV chamber (mbar) (×10^9^) as a function of temperature (K) measured by temperature-programmed desorption (TPD). 69 kGy γ-irradiated copper sample (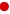); non-irradiated sample, background (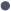). Heating rate is 6 K/min.

**Figure 4:**
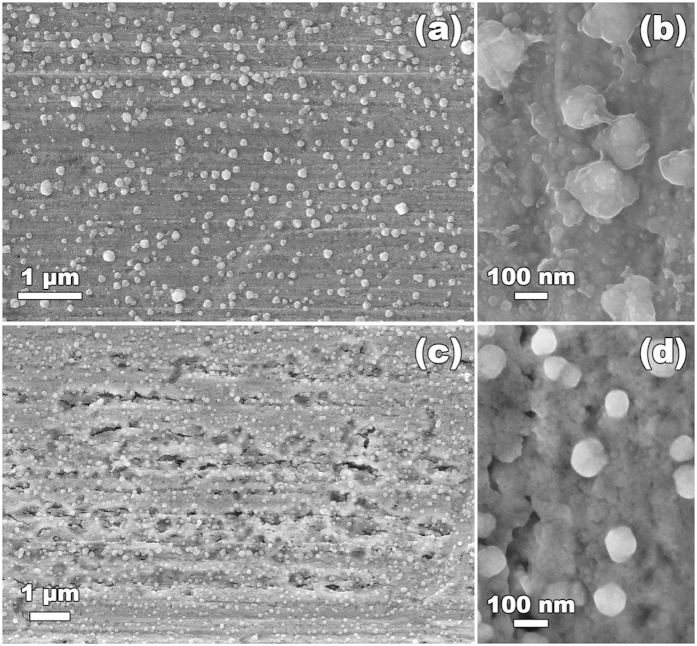
Particles formed at the surface of metallic Cu in oxygen-free water: (a,b) non-irradiated sample, (c,d) sample which was exposed to a total dose of γ-radiation of 69 kGy.

**Figure 5:**
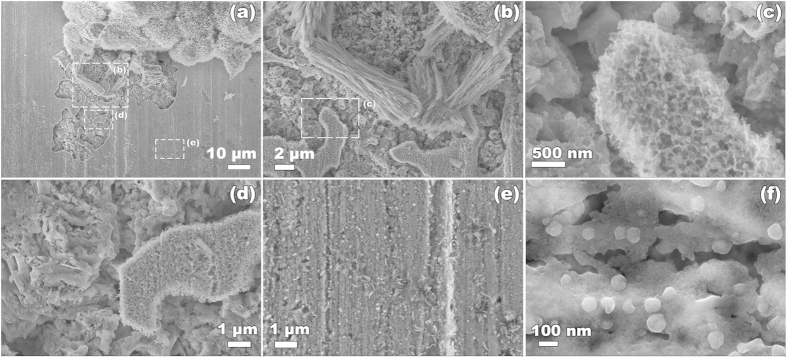
Scanning electron microscopy images of erosion at the surface of metallic copper kept in oxygen-free water and exposed to a radiation dose of 69 kGy. (**a–e**) An area next to an island of needle-shaped crystals; (**f**) particles at an area of the surface which is free of needle-shaped crystals.

**Figure 6:**
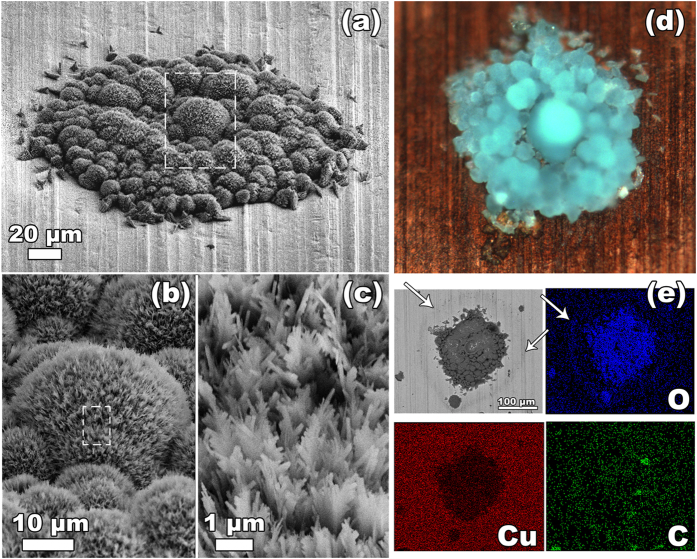
Islands of needle-shaped crystals formed at the surface of metallic copper in oxygen-free water after the γ-radiolysis (radiation dose of 69 kGy): (a–c) scanning electron microscopy images taken at different magnifications, (d) optical image, (e) scanning electron microscopy image (backscattered electrons) and corresponding energy dispersive X-ray maps obtained using K-lines of O, Cu and C.

The γ-radiation-induced hydrogen charging of copper was investigated as a function of total radiation dose (*D*). For each total radiation dose, blanks or background samples were prepared. The background samples consist of non-irradiated copper exposed to water for the same period of time as that of the irradiated samples. The background samples are necessary because copper has some amount of hydrogen from the manufacturing and even without the influence of ionizing radiation copper can induce the formation of minute amounts of H_2_(g) when exposed to water^37^,^38^. This is because water can adsorb dissociatively on some copper surfaces and this process leads to the formation of both HO^•^ and H^•^, the latter is a precursor of H_2_^38^. In Fig. 2 the amount of H_2_(g) measured in copper that was irradiated in aqueous media is plotted as a function of the total dose (*D*) corrected for the background values. It is known that desorption of dissociatively adsorbed water from metal and oxide surfaces can occur via a channel that leads to the formation of H_2_(g)^39^. This is due to the recombination of surface adsorbed H-atoms that occurs when the temperature is increased in the temperature-programmed desorption (TPD) experiment^40^. In order to exclude the hypothesis that the H_2_ measured in our samples originates from the recombination of products of dissociatively adsorbed water at the copper surface, we also determined the amount of H_2_O that is adsorbed at the surface of the copper samples and investigated possible correlations with the amount of H_2_ measured. In Fig. 2 it can be seen that the amount of H_2_O present in the copper samples does not correlate with the amount of H_2_ detected. This fact excludes the possibility that the H_2_ measured originates from the recombination of products of dissociatively adsorbed water at the copper surface. The H_2_(g) was measured post-irradiation which indicates that H^•^ and/or H_2_ are stable in copper. Previous investigations of desorption of H_2_ from copper show that bulk H_2_ desorbs in the same temperature range^41^ as the H_2_ that was detected in our irradiation experiments (Fig. 3). This indicates that the majority of the hydrogen measured originates from the copper bulk because surface-adsorbed hydrogen desorbs at lower temperatures in the TPD measurements. It can be seen in Fig. 3 that the irradiated sample has a considerably higher hydrogen content that desorbs at temperatures corresponding to bulk hydrogen, *T* > 500 K. The hydrogen content of the irradiated samples is also higher at the surface and at a few subsurface layers—this is for *T* < 500 K. A scanning electron microscopy investigation showed that the surfaces of both non-irradiated and irradiated samples exposed to water became covered with particles of approximately 100 nm in size (Fig. 4). There are no significant differences in morphology or size of the particles formed at the surface of non-irradiated and irradiated samples. However, besides the formation of nanoparticles, irradiated samples display extensive surface erosion and the presence of islands of needle-shaped crystals (Figs 4c,d, 5 and 6), which were not observed on non-irradiated samples. Erosion features are present all over the surface of the sample but are more pronounced in areas nearby islands of needle-shaped crystals (Fig. 5a–e). Here they reach considerable depth, affecting the material subsurface layers up to several hundred^33^ nm. As a result of erosion, a diversity of porous structures, cavities and ravines are formed (Fig. 5b–e). Needle-shaped crystals occur either as large islands (of approximately 50–200 μm) or as very small crystals surrounding eroded areas (Fig. 5e). Interestingly, all observed islands of needle-shaped crystals have a core part (central nucleation point (Fig. 6d)), which is surrounded by a circle of nucleation points consisting of smaller crystal islands that are in turn encircled by very small crystals (indicated by white arrows on Fig. 6e). Energy-dispersive X-ray microanalysis and X-ray photoelectron spectroscopy performed at these areas confirmed the presence of oxygen, copper and a small amount of carbon in the crystals (Fig. 6e). According to previous studies on the radiation chemistry of copper/water interfaces, the corrosion products formed are most likely oxygen-containing products—mostly cuprite, Cu_2_O—resulting from the oxidation of copper^33^. All islands of needle–shaped crystals have a light blue color (Fig. 6d). This suggests the presence of copper (II) hydroxide or copper (II) hydroxy carbonate because cuprite has a red-pink color^42^.

## Discussion

According to the experimental data available, at an external pressure of 1 atm of H_2_(g), dry copper absorbs very little hydrogen at room temperature—in the order of 3 × 10^−6^ ppm. Nevertheless, at a temperature of 1173 K, the uptake of hydrogen by copper increases to 40 ppm^43^,^44^. The driving force for this process has been attributed to the increase in the number of H-atoms at the Cu surface caused by the increase in temperature. This is because at higher temperatures a larger fraction of H_2_ is dissociated into H-atoms. The uptaken hydrogen, which diffuses into the bulk of Cu as H-atoms, has been determined to be trapped at defects such as Cu atom vacancies and dislocations^26^,^45^. This implies that a material with a higher concentration of such defects will have a higher capacity to uptake H from its surroundings. Electrochemistry has also been used^46^ for driving the uptake of hydrogen by Cu. In this process, hydrogen atoms are electrochemically produced on the copper surface resulting in a significant hydrogen uptake. A mechanism for this process has been proposed and the authors concluded that the surface properties are determinant for the diffusion of hydrogen into bulk copper.

Based on the observations presented above, it is plausible that the γ-radiation-induced hydrogen uptake presented here can be attributed to the radiolytic formation of hydrogen atoms. For γ-radiolysis of oxygen-free bulk water, the G-value (in μmol·J^−1^) for H^•^ is 0.06 and for H_2_ is 0.047, respectively^19^. However, as mentioned above, the interfacial G-value can be considerably higher. In the radiolytic system, the competing processes related with the uptake of H-atoms by copper are^19^,^47^,^48^:


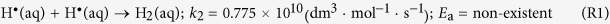



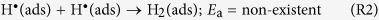



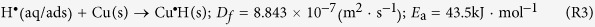


where Cu^•^H (s) denotes H-atoms dissolved in bulk copper, *D*_*f*_ is the diffusion coefficient for the H-atom in bulk polycrystalline copper and *k*_*2*_ is the second-order rate constant. R1 is fast, occurs in the diffusion limit and has no activation energy large enough that could allow its experimental determination. For H-atoms adsorbed at the Cu surface, R2 and R3 are competing processes. At a copper surface in contact with a gas-phase, R2 occurs without energy barrier at the (111) surface plane^49^. Yet, as highlighted by the authors of the cited study, the kinetics and energetics of such a process are dependent on the type of surface—the crystallographic plane—as well as the presence of surface defects. In aqueous media, R2 is expected to be slower—and have an energy barrier—due to the presence of surface defects and solvation effects as well as the hindering that these pose to the encounters between surface H-atoms when compared to the solid-gas interface^38^. The energy barrier for R3 corresponds to the transfer of an H-atom from solution into bulk Cu. This process is composed of several elementary steps that the H-atoms undergo and that occur in different time-scales: diffusion from solution to the interface; adsorption onto the Cu surface; and diffusion from the Cu surface into the bulk. The measured energy barrier of 43.5 kJ·mol^−1^ corresponds to the rate-determining step for the overall process described by reaction R3. For a surface adsorbed H-atom at a solid-liquid interface there is currently no kinetic data available in the literature for its diffusion onto the bulk Cu. Due to geometric factors, the growth of an irregular structure on the surface of Cu (Figs 5 and 6) can lead to a decrease in the yield of R2 and an increase in R3. This is because the oxygen-containing copper compounds formed on the metallic surface have a lower density and more defects than the metal surface. H-atoms adsorbed onto such irregular surfaces will have a lower probability for encounters when compared with the corresponding process occurring at the less defective metal surface. The rationale for this is that the H-atoms are more strongly bound to the irregular surfaces and thereby less mobile. Hence the rate constant for R2 is decreased. At the same time, the rate constant for R3 is enhanced for strongly bound H-atoms^50^. In conclusion, this will favor H-uptake by the solid material.

It should be noted that in the previous studies of hydrogen charging of copper, the availability of surface H_2_(g) was much higher than in the present case—because a pressure of H_2_(g) of 1 atm leads to an availability of hydrogen at the copper surface which is several orders of magnitude higher than that in the present radiolysis experiments^20^. In spite of this, the amount of H_2_(g) uptaken by copper at *T* = 298 K in the pressurized gas experiments^43^—3 × 10^−6^ wt.ppm at *p* = 1 atm of H_2_(g)—is 7 orders of magnitude lower than the amounts of H_2_(g) that were uptaken by the material due to exposure to γ-radiation in aqueous media at *T* = 298 K in the current study (Fig. 2). Additionally, in order for the pressurized gas experiments to be able to charge the same amounts of hydrogen into bulk Cu as we obtained in the radiolysis experiments, temperatures in the order of *T* ≈ 800 K have to be used together with an H_2_(g) external pressure of *p* = 1 atm. Our results clearly show that γ-radiation has a very large impact on the uptake of hydrogen by copper metal.

The γ-radiation induced uptake of hydrogen by bulk copper reported here most likely occurs due to a combination of several phenomena where the primary cause is the production of H-atoms at the interface due to γ-radiolysis of interfacial water. Additional phenomena to consider are the formation of defects in the bulk solid when it undergoes γ-radiation annealing and structural alterations of the surface and subsurface layers of copper enhancing the diffusion of H-atoms into the bulk. The insights presented here are not only essential for improving the radiation resistance of materials for long-term nuclear waste storage, but also for the design of novel hydrogen storage compounds and materials for space exploration.

## Methods

### Sample preparation and irradiation

All the solutions used in this study were prepared using water from a Millipore Milli-Q system. Samples of copper metal (Aldrich, 99.98%)—size: 5 × 5 × 0.25 mm—were subject to a surface treatment in order to remove traces of oxide, carbon dioxide and other possible contaminants. The samples were placed in an ultrasonic water bath for a period of 5 min and after removal were washed and transferred to a glove-box with an inert N_2_(g) atmosphere. Under the protective atmosphere the samples were washed with a solution of 5% amidosulfonic acid (H_3_NSO_3_) (Aldrich, 99.9%) during 1 min. It has been shown previously that the amidosulfonic acid is an effective way for removing the products of copper oxidation from the copper surface^51^. After washing in the acidic solution, the copper samples were further washed with ethanol and water, and placed in vials containing 5 ml of water, sealed and irradiated. γ-Irradiation was performed using a MDS Nordion 1000 Elite ^137^Cs γ-source with a dose rate of 0.135 Gy·s^−1^, which was determined by Fricke dosimetry^52^.

### Hydrogen measurements

The hydrogen content in the copper samples was determined using the thermal desorption spectroscopy (TDS) technique. The measurements were carried out with a thermal desorption apparatus designed and assembled at Aalto University, Finland. The apparatus consists of an ultra-high vacuum (UHV) chamber equipped with a vacuum furnace, coupled to a second air-lock vacuum chamber through which the samples are inserted. Additionally, there is a sample transportation mechanism with magnetic support for the sample basket. The pumping system has an effective pumping rate of 6.6 × 10^−2^ m^3^ s^−1^. The mass spectrometer unit and the furnace heating system are controlled with a computer using the Lab View software. The vacuum in the UHV chamber is kept at 7 × 10^−9^ bar. The heating system provides a direct control of the sample temperature in the temperature range from room temperature to 1123 K with a controlled heating rate that can be varied in the range 1–10 K·min^−1^. Prior to the TDS measurements, the samples were cleaned with acetone in an ultrasonic bath for 1 min and dried in a He gas flow to remove the residual water from the samples surface. The size of the samples for TDS measurements was 0.9 × 4.0 × 10.0 mm. The hydrogen concentration in the copper samples was determined from integration of the area of the TDS peaks after background correction.

### Scanning electron microscopy

For scanning electron microscopy experiments samples were taken from solution, dried with compressed air flow, placed onto stubs and immediately transferred to the microscope chamber, so that the total exposure of samples to air was less than 30 min. Images were collected using a Zeiss Gemini Ultra Plus scanning electron microscope and a FEI Quanta 3D Dual Beam system, equipped with an Oxford Instruments energy-dispersive detector.

## Additional Information

**How to cite this article**: Lousada, C. M. *et al.* Gamma radiation induces hydrogen absorption by copper in water. *Sci. Rep.* **6**, 24234; doi: 10.1038/srep24234 (2016).
